# A Neural Network Framework for Cognitive Bias

**DOI:** 10.3389/fpsyg.2018.01561

**Published:** 2018-09-03

**Authors:** Johan E. Korteling, Anne-Marie Brouwer, Alexander Toet

**Affiliations:** TNO Human Factors, Soesterberg, Netherlands

**Keywords:** cognitive biases, heuristics, decision making, rationality, information processing, neural networks, brain, neuroscience

## Abstract

Human decision-making shows systematic simplifications and deviations from the tenets of rationality (‘heuristics’) that may lead to suboptimal decisional outcomes (‘cognitive biases’). There are currently three prevailing theoretical perspectives on the origin of heuristics and cognitive biases: a cognitive-psychological, an ecological and an evolutionary perspective. However, these perspectives are mainly descriptive and none of them provides an overall explanatory framework for the underlying mechanisms of cognitive biases. To enhance our understanding of cognitive heuristics and biases we propose a neural network framework for cognitive biases, which explains why our brain systematically tends to default to heuristic (‘Type 1’) decision making. We argue that many cognitive biases arise from intrinsic brain mechanisms that are fundamental for the working of biological neural networks. To substantiate our viewpoint, we discern and explain four basic neural network principles: (1) Association, (2) Compatibility, (3) Retainment, and (4) Focus. These principles are inherent to (all) neural networks which were originally optimized to perform concrete biological, perceptual, and motor functions. They form the basis for our inclinations to associate and combine (unrelated) information, to prioritize information that is compatible with our present state (such as knowledge, opinions, and expectations), to retain given information that sometimes could better be ignored, and to focus on dominant information while ignoring relevant information that is not directly activated. The supposed mechanisms are complementary and not mutually exclusive. For different cognitive biases they may all contribute in varying degrees to distortion of information. The present viewpoint not only complements the earlier three viewpoints, but also provides a unifying and binding framework for many cognitive bias phenomena.

## Introduction

In daily life, we constantly make judgments and decisions (either conscious or unconscious) without knowing their outcome. We typically violate rules of logic and probability and resort to simple and near-optimal heuristic decision rules (‘mental shortcuts’) to optimize the likelihood of an acceptable outcome. This may be effective in conditions with time-constraints, lack or overload of relevant information, or when no optimal solution is evident (Simon, 1955; Gigerenzer and Gaissmaier, 2010). We are also inclined to use heuristics when problems appear familiar and when we do not feel the need to gather additional information. Heuristics can result in quite acceptable outcomes in everyday situations and when the time cost of reasoning are taken into account. However, people’s decisions may also deviate from the tenets of logic, calculation, and probability in ways that are inadvisable, leading to suboptimal decisions in terms of invested time and effort (costs) given the available information and expected benefits (Shafir and LeBoeuf, 2002). Rationality is here defined as decision making processes that are effective in achieving personal goals. This does not *necessarily* include the following of strict normative standards, such as the rules of logic and Bayesian probability (Elqayam and Evans, 2011). For example, the well-known confirmation bias may be motivated by argumentative reasons: as skilled arguers we may always be proactively looking for arguments that may well defend our opinions and/or that may persuade others instead of looking for the truth (Mercier and Sperber, 2011a,b). This means that argumentation and persuasion may in some cases be more effective for personal goals than truth. This matches the view of ‘soft normativism’ (e.g., Over, 2007; Stupple and Ball, 2014) purporting that evaluations of decision making quality do not necessarily require normative standards, but may well be enriched or enhanced by them. Having said this, in many cases, personally effective thinking would include logical or rational principles, like non-contradictory reasoning or focusing on, and appropriately weighting relevant information while ignoring irrelevant information. The suboptimal decisions that may result from heuristic decision making processes are known as ‘cognitive biases’ (Tversky and Kahneman, 1974). Using heuristics, we typically feel quite confident about our decisions and judgments, even when evidence is scarce and when we are aware of our cognitive inclinations (Risen, 2015). In line with this, specific cognitive biases are quite pervasive and persistent. Also, they are surprisingly systematic: in a wide range of different conditions, people tend to use similar heuristics and show the same cognitive biases (Shafir and LeBoeuf, 2002; Kahneman, 2011). This seemingly universal consistency calls for a generic explanation.

According to Lieder et al. (2017) the discovery of cognitive biases and the following doubt on human rationality shakes the foundations of economics, the social sciences and rational models of cognition. If human thinking does not show some internalized form of extensional, classical logic where it should conform to such a normative standard (Evans, 2002), human rationality is brought into question. If alternative normative systems are not available or arbitrary (Cohen, 1981; Dennett, 1989) there is little ground for deriving unifying laws of cognition from a set of basic axioms (Lieder et al., 2017). In our opinion, it is therefore important to gain more insight into the origins and underlying mechanisms of heuristics and cognitive biases. This may provide more guidance for how to explain and model cognitive processes in order to adequately understand and predict human behavior. In this regard, the literature presents three prevailing viewpoints on the origin of heuristics and cognitive biases: a cognitive-psychological perspective, an ecological perspective, and an evolutionary perspective. These three perspectives are complementary and emphasize different aspects of heuristic thinking. However, they provide no general explanation why cognitive biases occur and why they are so systematic, persistent, and consistent over different people and conditions. They also do not provide a framework with unifying underlying principles or mechanisms that are consistent with neuroscientific knowledge. These shortcomings have also stimulated an ongoing discussion in the literature on the extent to which human decision making reflects some rational or normative standard and whether or not it should be evaluated against such a system (e.g., Elqayam and Evans, 2011). However, until now this debate has not resulted in a set of axioms and principles that may form a basis for explaining the expressions of human cognition and (ir)rationality. To provide this guidance, we will outline a first framework of principles based on intrinsic brain characteristics, i.e., characteristics that are fundamental to biological neural networks.

## Current Perspectives on Cognitive Biases

The *cognitive-psychological* (or *heuristics and biases*) *perspective* (Evans, 2008; Kahneman and Klein, 2009) attributes cognitive biases to limitations in the available data and in the human information processing capacity (Simon, 1955; Broadbent, 1958; Kahneman, 1973, 2003; Norman and Bobrow, 1975). In this view, we tend to use simple heuristics in complex, unfamiliar, uncertain, and/or time-constrained situations because we can only process a limited amount of the available information (‘limited-’ or ‘bounded rationality’: Simon, 1955; Kahneman, 1973; Norman and Bobrow, 1975; Gigerenzer and Selten, 2002). This may produce quite acceptable outcomes, but decision errors or biases may occur when relevant information is ignored or inappropriately weighted and when irrelevant information interferes (Kahneman, 2003; Evans, 2008). This view has resulted in dual-process heuristic-deliberate frameworks that postulate a distinction between fast, intuitive, automatic, heuristic, and emotionally charged (heuristic, ‘System 1’ or ‘Type 1’) processes versus slow, conscious, controlled, deliberate and analytic (deliberate ‘System 2’ or ‘Type 2’) processes (Evans, 2008). Although the ‘Systems 1 and 2’ terminology (originally proposed by Stanovich and West, 2001) suggests concrete underlying and explanatory brain systems, this terminology was merely adopted for reasons of making it more easily comprehendible. When fast decisions are required, performance is based on low-effort heuristic processes. In complex or unfamiliar situations or when sufficient time is available, deliberate processes may monitor and revise the output of the (default) heuristic processing type (Evans, 1984, 1989; Kahneman, 2003, 2011). In this view, biases occur in these situations when deliberate processing either (1) fails to successfully engage (Kahneman, 2003) or (2) fails to override the biased heuristic response (Evans and Stanovich, 2013). The slower deliberate processes rely on time- and resource-consuming serial operations on the available data and are constrained by the limited capacity of the central working memory (Baddeley and Hitch, 1974; Baddeley, 1986; Evans and Stanovich, 2013). Conversely, heuristic processes do not demand executive working memory resources and operate implicitly, in parallel, and are highly accessible (Kahneman, 2003; De Neys, 2006).

The *ecological perspective* points out that heuristics can be quite effective in practical and natural situations (Gigerenzer, 2000; Goldstein and Gigerenzer, 2002; Gigerenzer and Gaissmaier, 2010). This perspective attributes cognitive biases to a mismatch between heuristics and the context or the environment in which they are applied (Klein, 1993, 1998, 2008). Biases occur when people apply experience-based heuristics (‘Naturalistic Decision Making’: Klein, 1993, 1998, 2008) in unknown or unfamiliar conditions that do not match their mental model, as is the case in experiments performed in most artificial- or laboratory settings, or when people lack relevant expertise. In this view, people use effective heuristics (acquired through learning or experience) that are based on the spatiotemporal regularities of the context and environment in which they routinely live and work (‘ecological rationality’). Only through extensive experience and dedicated learning can difficult cognitive tasks become more intuitive (Klein, 1998, 2008). A prerequisite for the development of adequate heuristics (i.e., for effective learning) is that the environment should be sufficiently stable and predictable, providing adequate feedback and a high number and variety of experiences (Shanteau, 1992; Klein, 1998). After sufficient training, experts will usually effectively rely on heuristic processing, but may switch to deliberate reasoning when they notice that they are relatively unfamiliar with a given issue (‘adaptive rationality’).

The *evolutionary perspective* attributes cognitive biases to a mismatch between evolutionarily developed heuristics (‘evolutionary rationality’: Haselton et al., 2009) and the current context or environment (Tooby and Cosmides, 2005). In this view, the same heuristics that optimized the chances of survival of our ancestors in their (natural) environment can lead to maladaptive (‘biased’) behavior when they are used in our current (artificial) settings. Heuristics that have been proposed as examples of this kind of mismatch are the action bias (a penchant for action even when there is no rational justification to deviate from the default option of no-action; Patt and Zeckhauser, 2000; Ashby et al., 2017), loss aversion (the disutility of giving up an object is greater than the utility associated with acquiring it: Kahneman and Tversky, 1984) and the scarcity heuristic (a tendency to attribute greater subjective value to items that are more difficult to acquire or in greater demand: Mittone and Savadori, 2009). While the evolutionary and ecological perspectives both emphasize that bias only occurs when there is a mismatch between heuristics and the situations in which they are applied, they differ in the hypothesized origin of this mismatch: the ecological perspective assumes that adequate heuristics are currently lacking or inappropriately applied for now, while the evolutionary perspective assumes that they are largely genetically determined. In both views, biases may also represent experimental artifacts that occur when problems (that can in principle be solved in a rational way) are presented in unusual or unnatural formats, as in many laboratory studies (Haselton et al., 2005, 2009).

While all three perspectives (or frameworks) on cognitive biases provide descriptions and insights concerning the tendency to deviate from strict rationality, they also have their limitations. For instance, limited capacity as a central explanatory construct may easily lead to rhetoric tautological explanations. Experimental findings of heuristics and bias are often ‘explained’ by limited capacity in processing resources, while this latter kind of capacity limitation is inferred from the empirical fact of heuristics and bias. This is a classical *circulus vitiosus*: what has to be explained (limited output capacity) is part of the explanatory construct (limited input capacity). The explanation of bias mainly involves a different and more generic phrasing of the problem, which is logically irrefutable (Neumann, 1987). It is difficult to predict when a given decision task sufficiently exceeds our cognitive capacity and will lead to cognitive bias. Similarly, concerning mismatch as an explanatory construct, it is hard to say beforehand when a problem differs sufficiently from a given context to induce biased reasoning. More importantly, limited capacity and mismatch do not explain why biases, such as superstition (Skinner, 1948; Risen, 2015), or overconfidence, persistently occur, even when we are (made) aware of them and when we have ample knowledge and time to do better (Kahneman, 2011; Risen, 2015). The psychological and ecological viewpoint also do not explain why many heuristics and biases are so *consistent* over many different people and situations, i.e., why people, independent of expertise and character, so systematically show the same, typical idiosyncrasies in reasoning- and judgment (e.g., Shafir and LeBoeuf, 2002; Kahneman, 2011). This similarity of heuristics seems in contradiction with the fact that in other kinds of tasks different people show a broad variety of approaches or working methods, which may result in many different kinds of errors or solutions when these tasks are (too) difficult, uncertain, or unfamiliar (Shafir and LeBoeuf, 2002). In addition, the current explanations are not always clear on why these consistencies in human decision making are so *specific* or *typical*. Why do we mostly incline to over-confidence instead of under-confidence, to confirmation instead of disconfirmation, or to neglect of (small) probabilities in some situations while over-valuating them in others (e.g., Kahneman, 2011)? Why do we make risk-averse choices if the expected outcome is positive, but make risk-seeking choices to avoid negative outcomes, i.e., the pseudo-certainty effect (Tversky and Kahneman, 1981b; Hardman, 2009). Furthermore, many explanations of cognitive biases are also rather specific and not easily generalized, in some cases even contradicting their own rationale. For instance, in some situations, heuristics and biases typically seem to include an *increase* rather than *decrease* in the amount of information that needs to be processed. Examples are the base rate neglect (specific information is preferred over general information: Tversky and Kahneman, 1982), the conjunction fallacy (a combination of conditions is considered more likely than only one of those conditions: Tversky and Kahneman, 1983), the story bias (consistent and believable stories are more easily accepted and remembered than simple facts: Turner, 1996; Dawes, 2001), and the representativeness bias (the probability that an entity belongs to a certain category is assessed by its similarity to the typical features of that category instead of its simple base rate: Tversky and Kahneman, 1974). Heuristics are also used and biases also occur in relatively *simple* and *obvious* decision situations without time pressure, such as in the pseudo-certainty effect (e.g., having trouble taking a loss on shares), the endowment effect (ascribing more value to items solely because you own them: Kahneman et al., 1990), superstition, the conjunction fallacy, the story bias, or the omission bias (the tendency to judge harmful actions as worse, or less moral, than equally harmful omissions or inactions: Baron and Ritov, 2004; Baron, 2008). Finally, biases are observed in the behavior of higher as well as lower animals (Sweis et al., 2018). Especially for animals, the literature shows many examples of bias (e.g., Dawkins and Brockmann, 1980; Chen et al., 2006; Lakshminaryanan et al., 2008). These animal biases are usually explained on the basis of evolutionary survival principles. But recently human-like biases have even been observed in a subset of artificial intelligence, called machine learning. Computer programs with similar properties as biological neural networks, such that they are able to “learn” from correlations and trends in the semantic and textual input without being explicitly programmed to do so (i.e., artificial neural networks) showed current human cultural stereotypes and prejudices (Caliskan et al., 2017). Apart from the operation of basic evolutionary mechanisms, it seems highly unlikely that animals show biases by developing human-like smart and practical (‘shortcut’) solutions in their decision making processes. The same holds for bias-like phenomena in artificial neural networks. Here, it should be noted that the aforementioned inconsistencies are generalist observations and probably not representative for all manifestations of bias, each of which always requires an analysis of the exact bias in that situation and how the issue is resolved depending on the amount, type, and quality of the information.^1^

Most of these objections concern the psychological and (to a lesser degree) the ecological explanations and only marginally the evolutionary perspective. In its present form the evolutionary perspective may explain many social psychological (and other survival-related) biases, such as group think, reciprocity, liking, or ingroup bias, whereas it only seems relevant for a limited number of purely *cognitive* biases. Finally, the multitude of (often phenomenologically described) biases that are often also quite similar (one being a specific example of the other) calls for a more unifying and binding framework of underlying mechanisms.

## A Neural Network Framework on Cognitive Biases

To enhance our understanding of cognitive heuristics and biases, we propose a *neural network* perspective that explains why our brain systematically tends to default to heuristic decision making. In this view, human decision making is determined by the basic design characteristics of neural information processing itself. These basic characteristics originally developed to perform concrete biological, perceptual, and motor functions which (almost) inevitably induce deviations from the abstract laws of logic and probability. With that we take explicit distance from the popular computer metaphor for human cognition, putting in its place a ‘neural network framework’ which is compatible with the way our brain works. This new framework builds on (and is consistent with) the basic biological and physiological principles constituting our brain as a biological neural network. It thereby not only complements the earlier three viewpoints, but provides also more fundamental and general insights for human cognitive behavior.

As Churchland (1987) remarked: “*The principal function of nervous systems is to get the body parts where they should be so that the organism may survive*.” The neural network framework elaborates (partly) on the evolutionary viewpoint by acknowledging that the functioning of our brain is based on universal mechanisms that helped us as a species to survive. During the evolution of (higher) animals, mechanisms that are typical for all biological neural networks (such as coincidence detection, facilitation, adaptation, and reciprocal inhibition) enabled the emergence of complex capabilities (e.g., motor skills, pattern recognition, and associative learning). These (Type 1) capabilities are essential to maintain our physical integrity (Damasio, 1994) in a (natural) environment (e.g., searching food, detecting danger, fight, or flight). However, they are very distinct from the “higher” cognitive functions like analytic and symbolic reasoning (Type 2) that developed much later from these ancient neural mechanisms. As formulated by Damasio (1994, p. 128): “*Nature appears to have built the apparatus of rationality not just on top of the apparatus of biological regulation, but also from it and with it.*” Despite their primal origin, perceptual-motor processes are highly complex and depend on the continuous parallel processing of massive incoming sensory data streams. The computational complexity of these processes becomes clear when we attempt to model and simulate them in logical machines (computers and robots). Our brain on the other hand, continuously and efficiently performs this kind of processing without any conscious effort. However, our brain is less optimized for (recently developed) cognitive functions that involve deliberate or analytic thinking (e.g., calculation, statistics, analysis, reasoning, abstraction, conceptual thinking) and that have only become essential for ‘survival’ in relatively modern civilizations. Our neural network framework conceives that biased decision making results from a mismatch between the original design characteristics of our brain as a neural network for performing perceptual-motor functions and maintaining biological integrity on the one hand and the nature of many conceptual or analytic problems on the other.

These original design characteristics determine or affect the way we use to solve cognitive problems, i.e., how we default to Type 1 thinking. For example, in cognitive deliberations we consistently put more weight to discrete jump-wise changes than to slow and gradual shifts, and we tend to judge phenomena on the basis of on relative differences and comparisons instead of on absolute values (‘contrast effect’). Relative changes are thus often seen as more important, or decisive, than absolute values. So we compare the price of a car radio with that of the whole car, and we put more effort for obtaining a discount of 1 euro on 5 than for a discount of 5 on 1,000. This tendency may originate from the fact that proportionality and relative differences dominate in human perception (e.g., the Weber–Fechner Law: Fechner, 1860 or Fitts’ law: Fitts, 1954). The mismatch between the nature of cognitive problems and perceptual-motor tasks for which the brain was originally optimized explains why we can easily and effortlessly perform computationally very complex perceptual-motor tasks (typically involving massively parallel data streams), whereas we are slow and experience great difficulty in solving Type 2 cognitive, logical or arithmetic problems that are computationally much more simple. It also explains why some heuristics and biases seem to include an *increase* in the amount and computational complexity (but not subjective difficulty) of information processing, rather than a *decrease*.

The neural network framework proposed here involves four basic principles that are characteristic for the working of biological neural networks. These basic mechanisms and characteristics of neural “wetware” (Kosslyn and Koenig, 1992) are inherent to (all) neural networks and therefore occur throughout the brain, that completely consists of large amounts of interconnected, assemblies of firing neurons. These principles are: (1) Association (Bar, 2007), (2) Compatibility (Thomson, 2000), (3) Retainment, and (4) *Focus*. Basically, these mechanisms – which will be discussed in more depth in the next sections – result in a modification (distortion or transformation) of the original or available data and its processing (e.g., weighting its importance). All neural networks typically include association (correlation) as their most fundamental underlying mechanism in all types of information processing (such as perception, cognition, memory, and motor skills). A substantial portion of the cognitive biases may originate from these associative properties. For example, lateral inhibition is an associative process resulting in the magnification of differences in neural activity (contrast enhancement), which is useful for perceptual-motor functions. However, for higher cortical functions, requiring exact calculation and proper weighting of data and the application of the rules of logic and probability, this transformation of data may work out detrimentally. As another example, selective weighting of information on the basis of the elapsed time or coincidental associations (based on coincidental similarities with irrelevant data) may hamper the formation of an well-balanced judgment about a situation, while this way of associative information processing is good for the execution of perceptual motor tasks. According to this viewpoint biases may be termed ‘hard wired.’ They can be seen as *cognitive illusions* originating from the same kind of underlying neural mechanisms that cause the many kinds of perceptual illusions (Reeves and Pinna, 2017).

So, the basic unifying construct of our framework is the ‘biological neural network’ that has ‘association’ as its most fundamental and basic ‘binding’ principle and three additional idiosyncrasies of neural networks that are inherently related to associative information processing. This forms the basis for our tendencies to (1) *associate* (unrelated) information, (2) to give priority to information that is *compatible and consistent* with our present knowledge, opinions, and expectations, (3) to *retain* given information that sometimes better could be ignored, and (4) to *focus* on dominant information while *neglecting* relevant information that is not directly available or recognized. In the next sections, we will discuss how each of these four default principles of neural information processing may contribute to the occurrence of biases in human cognition. While we do not claim to be able to discuss and relate each single reported bias to the four principles, we could identify four clusters of prominent cognitive biases that map rather straightforwardly on these principles. In order to illustrate the relation between neural principles and bias, we describe or mention examples of well-known heuristics and biases that are typical for the described principle. The supposed working principles are additional and not mutually exclusive. This means that specific biases may have a multifactor origin and thus may map to more than one principle. The four aforementioned principles help to establish a unifying and binding framework for the phenomenological collection of biases that are often quite similar, or one being a specific example of the other. In addition, they provide a link between the cognitive and the neuroscientific explanatory level. By bridging the gap between these explanatory levels, these concepts may help to understand how generic neural processes may lead to heuristic thinking and provide a starting point in neuroscientific and artificial neural network research.

### The Association Principle

The Association Principle states that (using correlation and coincidence detection) the brain ‘searches’ associatively for relationships, coherence, links, and patterns in the available information.

The brain (like all neural networks) functions in a highly associative way. Correlation and coincidence detection are the basic operations of neural functioning, as manifested in, e.g., Hebb’s rule (Hebb, 1949; Shatz, 1992), the ‘Law of Effect’ (Thorndike, 1927, 1933), Pavlovian conditioning (Pavlov, 2010), or autocorrelation (Reichardt, 1961). As a result, the brain automatically and subconsciously ‘searches’ for correlation, coherence, and (causal) connections: it is highly sensitive to consistent and invariant patterns. Association forms the basis of our unequaled pattern recognition capability, i.e., our ability to perceive coherent patterns and structures in the abundance of information that we absorb and process. The patterns we perceive can be based on many relationships, such as covariance, spatiotemporal coincidences, or similarity in form and content. Because of this associative way of perceiving, understanding, and predicting the world we tend to arrange our observations into regular, orderly relationships and patterns, and we have difficulty in dealing with randomness, unpredictability, and chaos. Even when these relations and patterns are accidental, our brain will be inclined to see them as meaningful, characteristic, and causally connected (Beitman, 2009). We associatively tend to classify events, objects or individuals into coherent categories that are determined by stereotypical combinations of features and traits. Examples of heuristics and bias resulting from associative information processing are the control illusion (people tend to overestimate the degree to which they are in control (Langer, 1975; Matute et al., 2015), superstition (Skinner, 1948; Risen, 2015), spurious causality (seeing causality in unconnected correlations), the conjunction fallacy (Tversky and Kahneman, 1983), the representativeness heuristic (Tversky and Kahneman, 1981a), and the previously mentioned story bias. We will now discuss the superstition bias (as an example of the association principle) in more detail to show how it can be explained in terms of neural mechanisms. Many people associate their success in a sports- or gambling game with whatever chance actions they performed immediately before winning. Subsequently they tend to repeat these same actions every time they play. This may even lead to widely shared rituals. A few accidental co-occurrences of a certain behavior and a favorable outcome may already suffice to induce and maintain this kind of behavior despite many subsequent contradicting instances. Performing their rituals tends to give people a feeling of control, even if there is no relation with the actual outcome of their following actions, e.g., the control illusion (Langer, 1975). According to Risen (2015) this ‘magical thinking’ is surprisingly common. In line with the Hebb doctrine (Hebb, 1949), the neural network framework contributes to an explanation of these phenomena by the way (the weight of) connections between neurons are affected by covarying inputs. Synaptic strengths are typically altered by either the temporal firing pattern of the presynaptic neuron or by modulatory neurons (Marder and Thirumalai, 2002). Neurons that repeatedly or persistently fire together, change each other’s excitability and synaptic connectivity (Destexhe and Marder, 2004). This basic principle, i.e., “*cells that fire together, wire together*” (Shatz, 1992), enables the continuous adaptation and construction of neural connections and associations based on simultaneous and covarying activations. This serves the formation of connections between functional subsystems based on covarying environmental inputs. This forms the basis of human learning processes, such as classical, operant, and perceptual-motor learning: relationships are discovered and captured in the connectionist characteristics of cells and synapses. This basic principle of neural information processing makes the brain sensitive to capitalize on correlating and covarying inputs (Gibson, 1966; Gibson, 1979), causing a strong tendency to associatively perceive coherence, (causal) relationships, and patterns, and to build neural connections based on these, even when they did not exist. In a similar way, this natural preference for patterns and coherence in the available information may explain the conjunction fallacy seen in probability judgments (Tversky and Kahneman, 1983). In perception, a stimulus usually becomes more certain with the amount of different (sources of) information that is coherently connected to it (Gibson, 1966, 1979). However, in cognitive judgments this natural perceptual tendency, or preference, for coherent patterns may result in more certain probability judgments because a combination of coherent conditions (“the dramatic increase in the oil price led to a 30% drop in oil consumption”) feels more likely than only one of those conditions (“The oil consumption decreased by 30%”).

### The Compatibility Principle

The Compatibility (or Consistency) Principle states that associations are highly determined by their compatibility (match, consistency, conformity) with the momentary state and connectionist properties of the neural network, such that we see, recognize, accept or prefer information according to its consistency with what we already know, understand, expect, and value.

The brain is not like a conventional repository or hard disk that can take up and store any information that is provided, almost indifferently of its characteristics. Instead, it is an associative network that requires new or additional information to be compliant or consistent with its existing state. What is associatively selected, processed, and integrated is not only determined by stimulus characteristics like the saliency of a target in its context, but also by the compatibility (match) with the brain’s momentary state and connectionist characteristics. Based on our competences and preconceptions (resulting from training and experience) we predominantly see what we expect to see. If a hammer is all you have, every problem resembles a nail. This principle of compatibility in neural information processing implies a compulsion to be consistent with what we already know, think or have done, resulting in a tendency to ignore or overlook relevant information because it does not match with our current behavior or mindset. The most well-known biases resulting from this principle are the confirmation bias (a tendency to search for, interpret, focus on and remember information in a way that *confirms* one’s preconceptions: Nickerson, 1998), the belief bias (the tendency to judge conclusions based on consistency with our prior beliefs, values, and knowledge rather than logical validity: e.g., Evans et al., 1983), and cognitive dissonance (a state of having inconsistent thoughts and a resulting tendency to search for and select consistent information: Festinger, 1957). Other examples of this kind of biased reasoning are the curse of knowledge (difficulty with taking the perspective of lesser-informed people: Kennedy, 1995), the familiarity heuristic (familiar items are favored over unfamiliar ones: Park and Lessig, 1981) and the sunk-cost fallacy (tendency to consistently continue a chosen course with negative outcomes rather than alter it: Arkes and Ayton, 1999). Elaborating on the confirmation bias as an example: When people perceive information, they tend to selectively notice examples that are consistent with (confirm) their existing (superstitious) intuitions. This may be explained by the fact that neural networks are more easily activated by stimulus patterns that are more congruent with their established connectionist properties or their current status. An example is priming (the exposure to one stimulus influences the response to another: Meyer and Schvaneveldt, 1971; Bao et al., 1997). For example, a word is more quickly and easily recognized after the presentation of a semantically related word. When a stimulus is experienced, subsequent experiences of the same stimulus will be processed more quickly by the brain (Forster and Davis, 1984). The situational context activates connected neural (knowledge) structures (characteristics, patterns, stereotypes, similarities, and associations) in an automatic and unconscious manner (Bargh et al., 1996). In general neural information processing is characterized by processes such as potentiation and facilitation (Katz and Miledi, 1968; Bao et al., 1997). Neural facilitation means that a post-synaptic action potential evoked by an impulse is increased when that impulse closely follows a prior one. This is caused by a residue of ‘active calcium’ entering the terminal axon membrane during the nerve impulse leading to an increased release of neurotransmitter (Katz and Miledi, 1968). Hence, a cell is activated more easily (its activation threshold is lower) directly after a prior activation. These, and other short-term synaptic changes support a variety of computations (Abbott and Regehr, 2004; Jackman and Regehr, 2017). Potentiation works on similar principles but on longer time scales (tens of seconds to minutes: Bao et al., 1997). Long-term Hebbian forms of plasticity such as potentiation make the processing of incoming information more efficient and effective when this information complies with previous activations (Destexhe and Marder, 2004; Wimmer and Shohamy, 2012). We suppose that these kinds of processes may form the basis for our tendency to interpret and focus on information that confirms previously established perceptions, interpretations or conclusions (i.e., the confirmation bias and the belief bias). So, whereas conventional repositories or hard disks can take up, process, and store information indifferently of its characteristics, in neural networks the selection and processing of inputs depends of the characteristics of the information. Compatible, conforming, or matching inputs are more easily selected, processed, and established, thus contributing to priming effects. This may explain why we see what we expect to see and why we associate more value or importance to information that aligns with what is already represented in our brains.

### The Retainment Principle

The Retainment Principle states that when irrelevant information or counter-productive information (which has been given before) is associatively integrated, it is captured in the brain’s neural circuitry, such that this cannot be simply made undone, erased, denied or ignored and thus will (associatively) affect a following judgment or decision.

While the brain associatively ‘searches’ for information that is consistent and compatible with its current state, it cannot completely disregard irrelevant, erroneous, or redundant information. For the brain, a hardware–software distinction does not apply: information is structurally encoded in ‘wetware’ (Kosslyn and Koenig, 1992). All stimuli entering the nervous system affect its physical–chemical structure and thereby its connectionist properties. So, unlike a computer program, once information has entered the brain, it cannot simply be ignored or put aside. It always has an effect. Consequently, it is nearly impossible to carry out an assignment like: “*Do not think of a pink elephant.*” Once perceived and processed by the brain, information is captured and retained and cannot easily be erased or ignored. This means that judgment and decision making is affected by persisting (‘anchoring’) effects of information that has been given and processed before the decision. Biased reasoning then occurs when irrelevant or misleading information associatively interferes with this process. Examples of this type of biased reasoning are the anchoring bias (decisions are biased toward previously acquired information or the ‘anchor’: Furnham and Boo, 2011), the endowment effect, the hindsight bias (the tendency to erroneously perceive events as inevitable or more likely once they have occurred: Hoffrage et al., 2000; Roese and Vohs, 2012), and the outcome bias (the perceived quality of a decision is based on its outcome rather than on the – mostly less obvious – factors that led to the decision: Baron and Hershey, 1988). The Hebbian principles of neural plasticity imply that the accumulation and processing of information necessarily causes synaptic changes, thereby altering the dynamics of neural networks (Destexhe and Marder, 2004). When existing circuits are associatively activated by new related inputs, their processing characteristics and outcomes (estimations, judgments, and decisions) will also be affected. This may be elucidated by the hindsight and outcome biases. Unlike conventional computer programs, the brain does not store new information independent and separately from old information. New information (an outcome of a previous decision) is associatively processed in existing circuits (‘memory’), which are consequently modified (through Hebbian learning). This neural reconsolidation of memory circuits, integrating new inputs with (related) existing representations will make the *exact representation* of the original information *principally* inaccessible for the brain. In behavioral terms: since hindsight or outcome knowledge is intrinsically connected to the memories about the original decision situation or event, new information received after the fact influences how the person remembers this original situation. Because of this blending of the neural representations of initial situations and outcomes the original representations must be reconstructed, which may cause a bias toward the final state. This may easily result in the tendency to see past events as having been predictable at the time they occurred, and in the tendency to weigh the ultimate outcome in judging the quality of a previous course of events (outcome bias). Likewise, the (long-term) possession of an item may result in more neural ingraining than something that is not (yet) owned. Loss of this property may then be more disruptive to the integrity of the associate neural circuitry than the idea of not acquiring this item. This may lead to the endowment effect, i.e., that people demand much more to give up an object than they would be willing to pay to acquire it.

### The Focus Principle

The Focus Principle states that the brain focusses associatively on dominant information, i.e., dominant ‘known knowns’ that easily pop up in the forming of judgments, ideas, and decisions. The fact that other (possible relevant) information may exist beyond is insufficiently recognized or ignored (like a blind spot).

When making a decision, the brain is not a logical system that systematically and proportionally takes into account and weighs all relevant information. Instead, our brain works more like a magnifying glass. When making decisions, we tend to rely on conclusions that are based on limited amounts of readily available information rather than on larger bodies of less consistent data (illusion of validity: Kahneman and Tversky, 1973). This overall tendency to overvalue a limited amount of information and to ignore other (weaker) sources brought Kahneman (2011) to the notion of: “*What you see is all there is*” (WYSIATI): only what pops up when making a decision ‘exists’; other information that may be useful but which is not directly available (i.e., the ‘*known unknowns*’ and the ‘*unknown unknowns*’), is (largely) ignored or not recognized. This ignorance of lacking information works in the opposite direction of the previously discussed Retainment Principle. This gap in our awareness of what we do *not* know resembles the *blind spot* in our visual field: the unaware obscuration of the visual field due to the absence of light-detecting photoreceptor cells on the location where the optic nerve passes through the optic disk of the retina. As result the corresponding part of the visual field (roughly 7.5° high and 5.5° wide) is not perceived. This local blindness usually remains completely unnoticed until one is subjected to specific tests (Wandell, 1995). A major example of this ‘blind spot’ phenomenon is the availability heuristic. This is the tendency to judge the frequency, importance or likelihood of an event by the ease with which relevant instances come to mind, which is highly determined by their imaginability or retrievability (Tversky and Kahneman, 1973, 1974). The availability bias causes us to ignore the impact of missing data: “*Out of sight, out of mind*” (Heuer, 2013). Also, we have a poor appreciation of the limits of our knowledge, and we usually tend to be overconfident about how much we already know. According to Kahneman (2011), the capacity of humans to believe the unbelievable on the basis of limited amounts of consistent information is in itself almost inconceivable (i.e., overconfidence bias). We often tend to be overconfident about the accuracy of our estimations or predictions (prognosis illusion) and we pay limited attention to factors that hamper the accuracy of our predictions (overconfidence effect: Moore and Healy, 2008). This is supposed to result from the fact that we do not see the unknowns (Kahneman, 2011) and that we have more information about ourselves than about others (Moore and Healy, 2008). Other examples of focus-biased reasoning are related to our overall tendency to focus on certain information while ignoring the rest, such as: the focusing illusion and focalism (the tendency to place too much emphasis on one or a limited number of aspects of an event or situation), the survivorship bias (a tendency to focus on the elements that survived a process and to forget about those that were eliminated: Brown et al., 1992), the priority heuristic (decisions are based on only one dominant piece of information: Brandstätter et al., 2006), the fundamental attribution error (explaining own behavior by external factors and other people’s behavior by personal factors: Ross, 1977), regression fallacy (failing to account for – relatively less visible – random fluctuation: Friedman, 1992), and the omission bias (a harmful action is usually more obvious, certain and clearly visible’ than a harmful inaction). An example of how the Focus principle in neural processes may explain our tendency to focus on and overvalue readily available information can be demonstrated by the way formerly established preconceptions may associatively enter a judgment and deliberation process and thus affect the resulting decision. In general, when people think about their superstitious intuitions, they are likely to automatically remember examples that support these. And when a compatible experience recently has reinforced such a superstitious belief, according to the Hebb rule it may be more easily activated again (compatibility). Moreover, these reconsolidating activations may also enhance inhibitory collateral outputs, which for example mediate the mechanism of lateral inhibition (Isaacson and Scanziani, 2011). Lateral inhibition in neural circuits involves a mutual inhibition of (competing) neurons proportionally to their activation level. In this way (groups of) neurons that are slightly more active can quickly become dominant. Lateral inhibition amplifies initially small differences (e.g., Mach Bands) and accelerates discriminatory processes leading to dominant associations (‘*The winner takes it all’*). These dominant associations may suppress the activation and retrieval of other (possibly contradicting) associations. This suppression may prevent that conflicting data or memories come to mind (‘blind spots’). It may also explain why we are unable to simultaneously perceive contradicting interpretations in ambiguous stimuli, like the Necker Cube. This basic neural mechanism of contrast enhancement by lateral inhibition makes us base our decisions on a limited amount of consistent information while we remain unware of the fact that we fail to consider additional relevant data or alternative interpretations. This way limited amounts of relatively strong ideas, habits or intuitions may easily dominate our decision-making processes by suppressing alternative but weaker processes.

## Discussion and Conclusion

Although our tendency to use heuristics that seem to violate the tenets of rationality generally leads to fairly acceptable outcomes with little experienced costs, it can sometimes result in suboptimal decisions. It is therefore difficult to conclude whether human decision making should generally be considered as ‘rational’ or not. This qualification depends on the context and on the chosen qualification standard. We chose not to go into the fundamental question whether human heuristic thinking should be considered as rational or irrational, but we focused on the origins and theoretical explanation of the pervasive and systematic character of heuristics and bias in human cognition. The result of this endeavor may provide axioms and principles to better explain and model cognitive processes in order to adequately understand human decision making.

The current three explanatory perspectives attribute biases in human decision making to cognitive capacity limitations and mismatches between available or deployed heuristics (that are optimized for specific conditions) and the conditions in which they are actually applied. As noted before, this does not explain why human decision making so universally and systematically violates the rules of logic and probability in relatively simple and obvious problem situations even when sufficient time is available, or why some heuristics and biases involve an increase in the amount of information processing (Shafir and LeBoeuf, 2002). This universal and pervasive character of heuristics and biases calls for a more fundamental and generic explanation. Based on biological and neuroscientific knowledge, we conjectured that cognitive heuristics and bias are inevitable tendencies linked to the inherent design characteristics of our brain. These characteristics are fundamental to the ‘wetware’ kind of associative functioning of biological neural networks (‘System 1’ or ‘Type 1’ processing) that originally developed to perform more basic physical, perceptual, and motor functions.

According to our framework, all neural networks typically include association as their most fundamental property. Our brain has a strong inclination or preference for coherence (i.e., pattern recognition and combining information on the basis of correlation and covariation). This tendency, which may be very efficient for the execution of perceptual-motor tasks, may lead to various distortions in the processing of cognitive information. Next to association, as the most fundamental and pervasive principle of neural wetware, we describe three additional characteristics that are directly related to associative information processing and that may affect decision making (i.e., Compatibility, Retainment, and Focus). The effects of the four discussed characteristics are additional and not mutually exclusive. So, the examples presented above should not suggest that there is a one-to-one relationship between underlying design principles of the brain and cognitive biases (Poldrack, 2010). Some biases even may have common origins that mutually reinforce each other. For example, on the basis of the strong preference of our brain for coherence (i.e., seeing coherent patterns or making combinations in the information on the basis of correlation and covariation) we tend to see combinations of unrelated information (i.e., superstition: Skinner, 1948). On top of that, this tendency may also have had survival value for our ancestors because it made them careful of any possible harmful coincidences. So this tendency of neural wetware to detect superstitious relationships may have been evolutionary reinforced.

All four principles may affect decision making and may contribute to cognitive biases, but the degree to which they do so may vary over different biases and situations. For example, the thoughts that come into our mind, or the information we consider when we make a decision (availability bias) always result from a continuing flow of *Associations*. Which (relevant or irrelevant) information is associatively included or preferred in our deliberations is affected by the characteristics of the given information in relation to our current neural state (*Compatibility*). The effects of (irrelevant) associative processes on our judgments or decisions cannot easily be suppressed or ignored (*Retainment*), and we focus on those thoughts that associatively do pop up, while *neglecting* relevant information that is not directly available or known (*Focus*). So, these intrinsic design characteristics form the basis for our inclinations to associate and combine (unrelated) information, to give priority to compatible information, to retain irrelevant information that should be ignored, and to focus on specific information, while ignoring possible relevant information that is not directly available or recognized. Although the number of heuristics and biases that have been identified in the psychological (and behavioral economics) literature is large, closer inspection reveals many similarities and consistencies among them, the one often being a specific example of the other. For example, biases and heuristics like conservatism, familiarity bias, recognition heuristic, confirmation bias, status quo bias, system justification, normalcy bias, illusion of truth, and the ‘not invented here’ bias all have in common our tendency to prefer what is compatible with (or conforms to) our present state. This abundance of often quite similar bias phenomena may be readily simplified and explained by the aforementioned unifying principles of neural networks. It should be noted, however, that it appeared not possible to relate the whole range (over 100) of bias phenomena to the four principles. The kind of biases we could not readily map onto the present four principles appeared to be those that are concerned with calculations and estimations on gain and loss and our poor abilities in statistical reasoning in general. So, the present framework does not readily explain why people seem not very concerned with the outcomes of probability reasoning (Kahneman and Tversky, 1979).

Our focus on the generic characteristics of neural information processing is not in conflict with the conception of the brain as an anatomically differentiated organ whose individual regions are functionally specialized and make specific contributions to mind and cognition (Finger, 2001). The notion that many parts and regions of the brain are associated with the expression of specific mental, behavioral, or cognitive operations is supported by a wealth of evidence from both anatomical and physiological studies, as well as from non-invasive neuroimaging. Functional specialization is one of the enduring theoretical foundations of cognitive neuroscience (van den Heuvel and Sporns, 2013). Our approach should be considered as complementary to the neuroscientific studies on functional specialization and interaction of brain regions. The intrinsic mechanisms and characteristics of neural processes that we propose are inherent to (all) neural networks and will therefore occur throughout the brain. So, our ‘generic’ approach of the issue is different from studies that relate specific brain regions and neural circuitry (e.g., limbic–frontal interactions) to cognitive phenomena, (Bechara et al., 1994, 1998; Miller et al., 2002; Barad et al., 2006; Fellows and Farah, 2007; Ghods-Sharifi et al., 2009; Yang and Raine, 2009; see also Goel et al., 2017). While we also link neural processes to biases and heuristics, this is done at level of connectionist properties of cell assemblies and the interaction between neurons in a network as this happens throughout the brain. It should thus be considered additional to the work on biases and heuristics in the functional brain anatomy sense. In general, it is likely that good choices are shaped by an interplay between cognitive and emotional processes, i.e., that both types of processing require prefrontal involvement as well as limbic involvement. However, according to our framework there is a strong, overall tendency to default to heuristic thinking that can be rather simply and readily explained by generic principles of neural wetware. Limbic–frontal interactions (Type 2) may be involved in modulating this pervasive default. When heuristics and biases emerge from the basic characteristics of biological neural networks, it is not very surprising that comparable cognitive phenomena are observed in animal behavior (e.g., Dawkins and Brockmann, 1980; Chen et al., 2006; Lakshminaryanan et al., 2008). For example, hyperbolic discounting has been found in rats, pigeons, and monkeys (Alexander and Brown, 2010). Also, in the domain of *artificial* neural networks, it has been found that applying standard machine learning to textual data results in human-like stereotyped biases that reflect everyday human culture and traditional semantic associations (Caliskan et al., 2017). Instead of supposing that cognitive strategies deal with decisional complexity, it seems likely that these kinds of findings reflect our conjectured relation between basic principles of neural networks and bias.

By identifying possible relations between ‘higher’ cognitive phenomena to established biological and neurophysiological processes, our neural network perspective may contribute to *cumulative* knowledge building, thus helping to bridge gaps between the cognitive- and neurosciences. Apart from clarifying the systematic and persistent nature of biases, and from resolving some theoretical contradictions (computational complexity of some heuristics) and limitations (*ad hoc* character of explanations), this framework may help to explain why only after extensive training domain-experienced decision makers become capable of solving difficult problems so easily and ‘intuitively.’ These experts do not analyze situations into their constituent components, nor do they explicitly calculate and weigh effects of different options (Type 2). Instead, their judgments and decisions seem to be based on the quick and automatic (Type 1) recognition of patterns in the available information (Klein, 1993, 1998). From this viewpoint prior experience and expertise (Klein, 1993, 1998) activate recognition (Simon, 1992, p. 155) or gut feelings (Gigerenzer, 2007) for the execution of complex decisions and actions. We provide more insights into the way this may work on a more basic neural level. According to our view heuristic (Type 1) processes originate from the generic characteristics of neural information processing that was originally aimed at pattern recognition, performing perceptual-motor tasks and maintaining physical integrity. With that our brain is not optimized for most higher-order cognitive functions, such as calculation, statistics and logical analysis. For this reason, only by extensive and consistent training with appropriate feedback the right neural associations can be established. These directly specify, or lead to, the right (unbiased) judgments and decisions, without the requirement for deliberation, much like the way neural processes for perceptual motor functions work. This makes domain-experienced experts capable of solving cognitive problems easily, instantaneously and ‘intuitively’ without any deliberation.

So far, a major conclusion of the present endeavor may be that it can be beneficial to adopt the conception of the brain as a *neural network* as the basis (or starting point) of psychological theory formation, instead of, e.g., the broadly (and often implicit) adopted computer metaphor. The realization of, and insight into, the *hard-wired* origins of biases may serve to find better ways to predict or counteract their occurrence. It may be this intrinsic character that makes our ‘automatic’ and ‘intuitive’ processes feel so overwhelming, pervasive and real that we often experience (emotional) reluctance to correct them (‘acquiescence’), irrespective of the awareness of our rational shortcomings (Risen, 2015). Since this affects almost all levels and components of public-, business-, and daily-life decision making, the understanding and mastering of biases is of great importance on many practical levels. It should be noted that, in view of the global character of the current psychological explanations, this paper provides a starting point for a more detailed (neural network) account of heuristics and biases. We hope this inspires further research in the areas of artificial networks and neuroscience.

## Author Contributions

JEK investigated the literature and wrote the article. A-MB contributed to the writing of the neuroscientific sections. AT searched the literature and critically reviewed the manuscript.

## Conflict of Interest Statement

The authors declare that the research was conducted in the absence of any commercial or financial relationships that could be construed as a potential conflict of interest.

## Footnotes

1 In combination with the intentions, knowledge, and skills of the subject.

## References

[B1] Abbott L. F. Regehr W. G. (2004). Synaptic computation. *Nature* 431 796–803. 10.1038/nature03010 15483601

[B2] Alexander W. H. Brown J. W. (2010). Hyperbolically discounted temporal difference learning. *Neural Comput.* 22 1511–1527. 10.1162/neco.2010.08-09-1080 20100071 PMC3005720

[B3] Arkes H. R. Ayton P. (1999). The sunk cost and Concorde effects: are humans less rational than lower animals? *Psychol. Bull.* 125 591–600. 10.1037/0033-2909.125.5.591

[B4] Ashby N. J. S. Rakow T. Yechiam E. (2017). ‘Tis better to choose and lose than to never choose at all. *Judgm. Decis. Mak.* 12 553–562.

[B5] Baddeley A. (1986). *Working Memory.* Oxford: Oxford University Press.

[B6] Baddeley A. D. Hitch G. (1974). “Working memory,” in *Psychology of Learning and Motivation*, ed. Bower G. H. (New York, NY: Academic Press), 47–89.

[B7] Bao J.-X. Kandel E. R. Hawkins R. D. (1997). Involvement of pre- and postsynaptic mechanisms in posttetanic potentiation at Aplysia synapses. *Science* 275 969–973. 10.1126/science.275.5302.969 9020078

[B8] Bar M. (2007). The proactive brain: using analogies and associations to generate predictions. *Trends Cogn. Sci.* 11 280–289. 10.1016/j.tics.2007.05.005 17548232

[B9] Barad M. Gean P.-W. Lutz B. (2006). The role of the amygdala in the extinction of conditioned fear. *Biol. Psychiatry* 60 322–328. 10.1016/j.biopsych.2006.05.029 16919522

[B10] Bargh J. A. Chen M. Burrows L. (1996). Automaticity of social behavior: direct effects of trait construct and stereotype activation on action. *J. Pers. Soc. Psychol.* 71 230–244. 10.1037/0022-3514.71.2.230 8765481

[B11] Baron J. (2008). *Thinking and Deciding*, 4th Edn, New York, NY: Cambridge University Press.

[B12] Baron J. Hershey J. C. (1988). Outcome bias in decision evaluation. *J. Pers. Soc. Psychol.* 54 569–579. 10.1037/0022-3514.54.4.569 3367280

[B13] Baron J. Ritov I. (2004). Omission bias, individual differences, and normality. *Organ. Behav. Hum. Decis. Process.* 94 74–85. 10.1016/j.obhdp.2004.03.003 26257616

[B14] Bechara A. Damasio A. R. Damasio H. Anderson S. W. (1994). Insensitivity to future consequences following damage to human prefrontal cortex. *Cognition* 50 7–15. 10.1016/0010-0277(94)90018-3 8039375

[B15] Bechara A. Damasio H. Tranel D. Anderson S. W. (1998). Dissociation of working memory from decision making within the human prefrontal cortex. *J. Neurosci.* 18 428–437. 10.1523/JNEUROSCI.18-01-00428.1998 9412519 PMC6793407

[B16] Beitman B. D. (2009). Brains seek patterns in coincidences. *Psychiatr. Ann.* 39 255–264. 10.3928/00485713-20090421-02

[B17] Brandstätter E. Gigerenzer G. Hertwig R. (2006). The priority heuristic: making choices without trade-offs. *Psychol. Rev.* 113 409–432. 10.1037/0033-295X.113.2.409 16637767 PMC2891015

[B18] Broadbent B. E. (1958). *Perception and Communication.* New York, NY: Pergamon Press 10.1037/10037-000

[B19] Brown S. J. Goetzmann W. Ibbotson R. G. Ross S. A. (1992). Survivorship bias in performance studies. *Rev. Financ. Stud.* 5 553–580. 10.1093/rfs/5.4.553

[B20] Caliskan A. Bryson J. J. Narayanan A. (2017). Semantics derived automatically from language corpora contain human-like biases. *Science* 356 183–186. 10.1126/science.aal4230 28408601

[B21] Chen M. K. Lakshminarayanan V. Santos L. R. (2006). How basic are behavioral biases? Evidence from capuchin monkey trading behavior. *J. Polit. Econ.* 114 517–537. 10.1086/503550

[B22] Churchland P. S. (1987). Epistemology in the age of neuroscience. *J. Philos.* 84 544–553. 10.5840/jphil1987841026

[B23] Cohen L. J. (1981). Can human rationality be demonstrated experimentally. *Behav. Brain Sci.* 4 317–370. 10.1017/S0140525X00009092

[B24] Damasio A. R. (1994). *Descartes’ Error: Emotion, Reason and the Human Brain.* New York, NY: G. P. Putnam’s Sons.

[B25] Dawes R. M. (2001). *Everyday Irrationality: How Pseudo-Scientists, Lunatics, and the Rest of Us Systematically Fail to Think Rationally.* Boulder, CO: Westview Press.

[B26] Dawkins R. Brockmann H. J. (1980). Do digger wasps commit the Concorde fallacy? *Anim. Behav.* 28 892–896. 10.1016/S0003-3472(80)80149-7

[B27] De Neys W. (2006). Automatic - heuristic and executive - analytic processing during reasoning: chronometric and dual-task considerations. *Q. J. Exp. Psychol.* 59 1070–1100. 10.1080/02724980543000123 16885144

[B28] Dennett D. C. (1989). *The Intentional Stance.* Cambridge, MA: MIT Press.

[B29] Destexhe A. Marder E. (2004). Plasticity in single neuron and circuit computations. *Nature* 431 789–795. 10.1038/nature03011 15483600

[B30] Elqayam S. Evans J. S. (2011). Subtracting “ought” from “is”: descriptivism versus normativism in the study of human thinking. *Behav. Brain Sci.* 34 233–248. 10.1017/S0140525X1100001X 22000212

[B31] Evans J. S. (2008). Dual-processing accounts of reasoning, judgment, and social cognition. *Annu. Rev. Psychol.* 59 255–278. 10.1146/annurev.psych.59.103006.093629 18154502

[B32] Evans J. S. Barston J. Pollard P. (1983). On the conflict between logic and belief in syllogistic reasoning. *Mem. Cogn.* 11 295–306. 10.3758/BF03196976 6621345

[B33] Evans J. S. B. T. (1984). Heuristic and analytic processes in reasoning. *Br. J. Psychol.* 75 451–468. 10.1111/j.2044-8295.1984.tb01915.x

[B34] Evans J. S. B. T. (1989). *Bias in Human Reasoning: Causes and Consequences.* London: Lawrence Erlbaum Associates, Inc.

[B35] Evans J. S. B. T. (2002). Logic and human reasoning: an assessment of the deduction paradigm. *Psychol. Bull.* 128 978–996. 10.1037/0033-2909.128.6.978 12405140

[B36] Evans J. S. B. T. Stanovich K. E. (2013). Dual-process theories of higher cognition: advancing the debate. *Perspect. Psychol. Sci.* 8 223–241. 10.1177/1745691612460685 26172965

[B37] Fechner G. T. (1860). *Elemente Der Psychophysik.* Leipzig: Breitkopf und Härtel.

[B38] Fellows L. K. Farah M. J. (2007). The role of ventromedial prefrontal cortex in decision making: judgment under uncertainty or judgment per se? *Cereb. Cortex* 17 2669–2674. 10.1093/cercor/bhl176 17259643

[B39] Festinger L. (1957). *A Theory of Cognitive Dissonance.* Stanford, CA: Stanford University Press.

[B40] Finger S. (2001). *Origins of Neuroscience: A History of Explorations into Brain Function.* New York, NY: Oxford University Press.

[B41] Fitts P. M. (1954). The information capacity of the human motor system in controlling the amplitude of movement. *J. Exp. Psychol.* 47 381–391. 10.1037/h0055392 13174710

[B42] Forster K. I. Davis C. (1984). Repetition priming and frequency attenuation in lexical access. *J. Exp. Psychol. Learn. Mem. Cogn.* 10 680–698. 10.1037/0278-7393.10.4.680

[B43] Friedman M. (1992). Do old fallacies ever die? *J. Econ. Lit.* 30 2129–2132.

[B44] Furnham A. Boo H. C. (2011). A literature review of the anchoring effect. *J. Socio Econ.* 40 35–42. 10.1016/j.socec.2010.10.008

[B45] Ghods-Sharifi S. St. Onge J. R. Floresco S. B. (2009). Fundamental contribution by the basolateral amygdala to different forms of decision making. *J. Neurosci.* 29 5251–5259. 10.1523/jneurosci.0315-09.2009 19386921 PMC6665478

[B46] Gibson J. J. (1966). *The Senses Considered As Perceptual Systems.* Oxford: Houghton Mifflin.

[B47] Gibson J. J. (1979). *The Ecological Approach To Visual Perception.* Boston, NJ: Houghton Mifflin.

[B48] Gigerenzer G. (2000). *Adaptive Thinking: Rationality in the Real World.* Oxford: Oxford University Press.

[B49] Gigerenzer G. (2007). *Gut Feelings: The Intelligence of the Unconscious.* New York, NY: Viking Press.

[B50] Gigerenzer G. Gaissmaier W. (2010). Heuristic decision making. *Annu. Rev. Psychol.* 62 451–482. 10.1146/annurev-psych-120709-145346 21126183

[B51] Gigerenzer G. Selten R. (2002). *Bounded Rationality: The Adaptive Toolbox.* Cambridge, MA: MIT Press.

[B52] Goel V. Navarrete G. Noveck I. A. Prado J. A. (2017). *The Reasoning Brain: The Interplay Between Cognitive Neuroscience and Theories of Reasoning.* Lausanne: Frontiers Media 10.3389/978-2-88945-118-0 PMC5214378 28105010

[B53] Goldstein D. G. Gigerenzer G. (2002). Models of ecological rationality: the recognition heuristic. *Psychol. Rev.* 109 75–90. 10.1037//0033-295X.109.1.75 11863042

[B54] Hardman D. (2009). *Judgment and Decision Making: Psychological Perspectives.* Leicester: Blackwell Publishing.

[B55] Haselton M. G. Bryant G. A. Wilke A. Frederick D. A. Galperin A. Frankenhuis W. E. (2009). Adaptive rationality: an evolutionary perspective on cognitive bias. *Soc. Cogn.* 27 733–762. 10.1521/soco.2009.27.5.733

[B56] Haselton M. G. Nettle D. Andrews P. W. (2005). “The evolution of cognitive bias,” in *The Handbook of Evolutionary Psychology*, ed. Buss D. M. (Hoboken, NJ: John Wiley & Sons Inc), 724–746.

[B57] Hebb D. O. (1949). *The Organization of Behavior.* New York, NY: Wiley.

[B58] Heuer R. J. (2013). “Cognitive factors in deception and counter deception,” in *The Art and Science of Military Deception*, eds Rothstein H. Whaley B. (Boston, MA: Artech House), 105–133.

[B59] Hoffrage U. Hertwig R. Gigerenzer G. (2000). Hindsight bias: a by-product of knowledge updating? *J. Exp. Psychol. Learn. Mem. Cogn.* 26 566–581. 10.1037/0278-7393.26.3.566 10855418

[B60] Isaacson J. S. Scanziani M. (2011). How inhibition shapes cortical activity. *Neuron* 72 231–243. 10.1016/j.neuron.2011.09.027 22017986 PMC3236361

[B61] Jackman S. L. Regehr W. G. (2017). The mechanisms and functions of synaptic facilitation. *Neuron* 94 447–464. 10.1016/j.neuron.2017.02.047 28472650 PMC5865607

[B62] Kahneman D. (1973). *Attention and Effort.* Englewood Cliffs, NJ: Prentice-Hall Inc.

[B63] Kahneman D. (2003). A perspective on judgment and choice: mapping bounded rationality. *Am. Psychol.* 58 697–720. 10.1037/0003-066X.58.9.697 14584987

[B64] Kahneman D. (2011). *Thinking, Fast and Slow.* New York, NY: Farrar, Straus and Giroux.

[B65] Kahneman D. Klein G. (2009). Conditions for intuitive expertise: a failure to disagree. *Am. Psychol.* 64 515–526. 10.1037/a0016755 19739881

[B66] Kahneman D. Knetsch J. L. Thaler R. H. (1990). Experimental tests of the endowment effect and the Coase theorem. *J. Polit. Econ.* 98 1325–1348. 10.1086/261737

[B67] Kahneman D. Tversky A. (1973). On the psychology of prediction. *Psychol. Rev.* 80 237–251. 10.1037/h0034747

[B68] Kahneman D. Tversky A. (1979). Prospect theory: an analysis of decision under risk. *Econometrica* 47 263–292. 10.1007/s11336-014-9425-x 25142256

[B69] Kahneman D. Tversky A. (1984). Choices, values, and frames. *Am. Psychol.* 39 341–350. 10.1037/0003-066X.39.4.341

[B70] Katz B. Miledi R. (1968). The role of calcium in neuromuscular facilitation. *J. Physiol.* 195 481–492. 10.1113/jphysiol.1968.sp008469 4296699 PMC1351674

[B71] Kennedy J. (1995). Debiasing the curse of knowledge in audit judgment. *Account. Rev.* 70 249–273.

[B72] Klein G. (1998). *Sources of Power: How People Make Decisions.* Cambridge, MA: MIT Press.

[B73] Klein G. (2008). Naturalistic decision making. *Hum. Factors* 50 456–460. 10.1518/001872008X288385 18689053

[B74] Klein G. A. (1993). “A recognition-primed decision (RPD) model of rapid decision making,” in *Decision Making in action: Models and Methods*, eds Klein G. A. Orasanu J. Calderwood R. Zsambok C. E. (Norwood, NJ: Ablex Publishing Corporation), 138–147.

[B75] Kosslyn S. M. Koenig O. (1992). *Wet Mind: The New Cognitive Neuroscience.* New York, NY: The Free Press.

[B76] Lakshminaryanan V. Chen M. Santos L. R. (2008). Endowment effect in capuchin monkeys. *Philos. Trans. R. Soc. B Biol. Sci.* 363 3837–3844. 10.1098/rstb.2008.0149 18840573 PMC2581778

[B77] Langer E. J. (1975). The illusion of control. *J. Pers. Soc. Psychol.* 32 311–328. 10.1037/0022-3514.32.2.311

[B78] Lieder F. Griffiths T. Huys Q. J. Goodman N. D. (2017). The anchoring bias reflects rational use of cognitive resources. *Psychon. Bull. Rev.* 25 322–349. 10.3758/s13423-017-1286-8 28484952

[B79] Marder E. Thirumalai V. (2002). Cellular, synaptic and network effects of neuromodulation. *Neural Netw.* 15 479–493. 10.1016/S0893-6080(02)00043-6 12371506

[B80] Matute H. Blanco F. Yarritu I. Díaz-Lago M. Vadillo M. A. Barberia I. (2015). Illusions of causality: how they bias our everyday thinking and how they could be reduced. *Front. Psychol.* 6:888. 10.3389/fpsyg.2015.00888 26191014 PMC4488611

[B81] Mercier H. Sperber D. (2011a). Argumentation: its adaptiveness and efficacy. *Behav. Brain Sci.* 34 94–111. 10.1017/S0140525X10003031 21447233

[B82] Mercier H. Sperber D. (2011b). Why do humans reason? Arguments for an argumentative theory. *Behav. Brain Sci.* 34 57–74. 10.1017/S0140525X10000968 21447233

[B83] Meyer D. E. Schvaneveldt R. W. (1971). Facilitation in recognizing pairs of words: evidence of a dependence between retrieval operations. *J. Exp. Psychol.* 90 227–234. 10.1037/h0031564 5134329

[B84] Miller E. K. Freedman D. J. Wallis J. D. (2002). The prefrontal cortex: categories, concepts and cognition. *Philos. Trans. R. Soc. Lond. B Biol. Sci.* 357 1123–1136. 10.1098/rstb.2002.1099 12217179 PMC1693009

[B85] Mittone L. Savadori L. (2009). The scarcity bias. *Appl. Psychol.* 58 453–468. 10.1111/j.1464-0597.2009.00401.x

[B86] Moore D. A. Healy P. J. (2008). The trouble with overconfidence. *Psychol. Rev.* 115 502–517. 10.1037/0033-295X.115.2.502 18426301

[B87] Neumann O. (1987). “Beyond capacity: a functional view of attention,” in *Perspectives on Perception and Action*, eds Heuer H. Sanders A. F. (Hillsdale, NJ: Lawrence Erlbaum), 361–394.

[B88] Nickerson R. S. (1998). Confirmation bias: a ubiquitous phenomenon in many guises. *Rev. Gen. Psychol.* 2 175–220. 10.1037/1089-2680.2.2.175

[B89] Norman D. A. Bobrow D. G. (1975). On data-limited and resource-limited processes. *Cogn. Psychol.* 7 44–64. 10.1016/0010-0285(75)90004-3 6214603

[B90] Over D. E. (2007). “Content-independent conditional inference,” in *Integrating the Mind: Domain General Versus Domain Specific Processes in Higher Cognition*, ed. Roberts M. J. (New York, NY: Psychology Press), 83–103.

[B91] Park C. W. Lessig V. P. (1981). Familiarity and its impact on consumer decision biases and heuristics. *J. Consum. Res.* 8 223–230. 10.1086/208859

[B92] Patt A. Zeckhauser R. (2000). Action bias and environmental decisions. *J. Risk Uncertain.* 21 45–72. 10.1023/a:1026517309871

[B93] Pavlov P. I. (2010). Conditioned reflexes: an investigation of the physiological activity of the cerebral cortex. *Ann. Neurosci.* 17 136–141. 10.5214/ans.0972-7531.1017309 25205891 PMC4116985

[B94] Poldrack R. A. (2010). Mapping mental function to brain structure: how can cognitive neuroimaging succeed? *Perspect. Psychol. Sci.* 5 753–761. 10.1177/1745691610388777 25076977 PMC4112478

[B95] Reeves A. Pinna B. (2017). The future of perceptual illusions: from phenomenology to neuroscience. *Front. Hum. Neurosci.* 11:9. 10.3389/fnhum.2017.00009 28217087 PMC5289987

[B96] Reichardt W. A. (1961). “Autocorrelation, a principle for the evaluation of sensory information by the central nervous system”, in *Sensory Communication*, ed. Rosenblith W. A. (New York, NY: Wiley), 303–317. 10.7551/mitpress/9780262518420.003.0017

[B97] Risen J. L. (2015). Believing what we do not believe: acquiescence to superstitious beliefs and other powerful intuitions. *Psychol. Rev.* 123 128–207. 10.1037/rev0000017 26479707

[B98] Roese N. J. Vohs K. D. (2012). Hindsight bias. *Perspect. Psychol. Sci.* 7 411–426. 10.1177/1745691612454303 26168501

[B99] Ross L. (1977). “The intuitive psychologist and his shortcomings: distortions in the attribution process,” in *Advances in Experimental Social Psychology*, ed. Leonard B. (New York, NY: Academic Press), 173–220.

[B100] Shafir E. LeBoeuf R. A. (2002). Rationality. *Annu. Rev. Psychol.* 53 491–517. 10.1146/annurev.psych.53.100901.135213 11752494

[B101] Shanteau J. (1992). Competence in experts: the role of task characteristics. *Organ. Behav. Hum. Decis. Process.* 53 252–266. 10.1016/0749-5978(92)90064-E

[B102] Shatz C. J. (1992). The developing brain. *Sci. Am.* 267 60–67. 10.1038/scientificamerican0992-60 1502524

[B103] Simon H. A. (1955). A behavioral model of rational choice. *Q. J. Econ.* 69 99–118. 10.2307/1884852

[B104] Simon H. A. (1992). What is an “Explanation” of Behavior? *Psychol. Sci.* 3 150–161. 10.1111/j.1467-9280.1992.tb00017.x

[B105] Skinner B. F. (1948). ‘Superstition’ in the pigeon. *J. Exp. Psychol.* 38 168–172. 10.1037/h0055873 18913665

[B106] Stanovich K. E. West R. F. (2001). Individual differences in reasoning: implications for the rationality debate? *Behav. Brain Sci.* 23 645–665. 10.1017/S0140525X00003435 11301544

[B107] Stupple E. J. Ball L. J. (2014). The intersection between Descriptivism and Meliorism in reasoning research: further proposals in support of ‘soft normativism’. *Front. Psychol.* 5:1269. 10.3389/fpsyg.2014.01269 25414687 PMC4220629

[B108] Sweis B. M. Abram S. V. Schmidt B. J. Seeland K. D. MacDonals A. W. Thomas M. J. (2018). Sensitivity to “sunk costs” in mice, rats an humans. *Science* 361 178–181. 10.1126/science.aar8644 30002252 PMC6377599

[B109] Thomson A. M. (2000). Facilitation, augmentation and potentiation at central synapses. *Trends Neurosci.* 23 305–312. 10.1016/S0166-2236(00)01580-0 10856940

[B110] Thorndike E. L. (1927). The law of effect. *Am. J. Psychol.* 39 212–222. 10.2307/1415413

[B111] Thorndike E. L. (1933). A proof of the law of effect. *Science* 77 173–175. 10.1126/science.77.1989.173-a 17819705

[B112] Tooby J. Cosmides L. (2005). “Conceptual foundations of evolutionary psychology,” in *Handbook of Evolutionary Psychology*, ed. Buss D. M. (Hoboken, NJ: John Wiley & Sons, Inc.), 5–67.

[B113] Turner M. (1996). *The Literary Mind: The Origins of Thought and Language.* Oxford: Oxford University Press.

[B114] Tversky A. Kahneman D. (1973). Availability: a heuristic for judging frequency and probability. *Cogn. Psychol.* 5 207–232. 10.1016/0010-0285(73)90033-9

[B115] Tversky A. Kahneman D. (1974). Judgment under uncertainty: heuristics and biases. *Science* 185 1124–1131. 10.1126/science.185.4157.1124 17835457

[B116] Tversky A. Kahneman D. (1981a). “Judgments of and by representativeness,” in *Judgment Under Uncertainty: Heuristics and Biases*, eds Kahneman D. Slovic P. Tversky A. (Cambridge: Cambridge University Press). 10.21236/ADA099502

[B117] Tversky A. Kahneman D. (1981b). The framing of decisions and the psychology of choice. *Science* 211 453–458. 10.1126/science.7455683 7455683

[B118] Tversky A. Kahneman D. (1982). “Evidential impact of base rates,” in *Judgment Under Uncertainty: Heuristics and Biases*, eds Kahneman D. Slovic P. Tversky A. (Cambridge: Cambridge University Press).

[B119] Tversky A. Kahneman D. (1983). Extensional versus intuitive reasoning: the conjunction fallacy in probability judgment. *Psychol. Rev.* 90 293–315. 10.1037/0033-295X.90.4.293 26635712 PMC4658440

[B120] van den Heuvel M. P. Sporns O. (2013). Network hubs in the human brain. *Trends Cogn. Sci.* 17 683–696. 10.1016/j.tics.2013.09.012 24231140

[B121] Wandell B. A. (1995). *Foundations of Vision.* Sunderland, MA: Sinauer Associates.

[B122] Wimmer G. E. Shohamy D. (2012). Preference by association: how memory mechanisms in the hippocampus bias decisions. *Science* 338 270–273. 10.1126/science.1223252 23066083

[B123] Yang Y. Raine A. (2009). Prefrontal structural and functional brain imaging findings in antisocial, violent, and psychopathic individuals: a meta-analysis. *Psychiatry Res. Neuroimag.* 174 81–88. 10.1016/j.pscychresns.2009.03.012 19833485 PMC2784035

